# Association between occlusal features and masticatory function in Hong Kong preschool children: a survey with one-year longitudinal follow-up

**DOI:** 10.1186/s12903-024-03895-6

**Published:** 2024-02-05

**Authors:** King Sang Rita Au Yeung, Zhiyi Shan, Fung Hou Kumoi Mineaki Howard Sum, Ka Wai Frank Wong, Hui Man Gillian Lee, Yanqi Yang

**Affiliations:** Division of Paediatric Dentistry and Orthodontics, Faculty of Dentistry, University of Hong Kong, Hong Kong, China

**Keywords:** Mastication, Malocclusion, Sucking behaviour, Dental caries, Preschool children

## Abstract

**Background:**

Mastication is important for breaking down food, aiding swallowing and nutrients absorption, and is therefore fundamental to a child’s development. Studies have shown poor masticatory function to be associated with younger age and presence of caries. However, studies of the association between masticatory function and malocclusion yielded contradictory results. The aim of this study is therefore to investigate the association between three-dimensional occlusal features with masticatory function, among preschool children in Hong Kong.

**Methods:**

Self-administered questionnaires on masticatory function in three domains, namely general chewing difficulty, requiring help when eating different food types and increased preference for soft food were completed by parents. Information on non-nutritive sucking habits and basic demographics were also collected in the questionnaire. Clinical examinations were conducted to record three-dimensional occlusal features and presence of caries. Baseline investigations and one-year follow-ups were undertaken for 1,566 and 996 preschool children. Association of poor masticatory function with occlusal features, sucking habits and caries was investigated using chi-squared tests. Binomial logistic regressions were then carried out incorporating any significant factors identified. Longitudinal analysis of the one-year follow-up data was carried out to investigate whether improved occlusal features, sucking habits and caries resulted in better masticatory function.

**Results:**

In the cross-sectional study, the first domain of general chewing difficulty was associated with caries and thumb/digit sucking. The second domain of requiring help when eating different food types was associated with the male sex, younger age, caries and pacifier use. The last domain of increased preference for soft foods was associated with caries and thumb/digit sucking. Occlusal features, including abnormal overjet and unilateral permanent molars not in contact, were significantly associated with poor masticatory function in the bivariate analyses, but were not significant in the logistic regressions. In the longitudinal analysis, general chewing difficulty was found to improve in those of older age and those with resolved anterior crossbite. Less help was required to eat meat in those with fewer caries. Similarly, less help was required to eat food containing bones in those with reduced pacifier use. Preferences for eating soft foods was reduced in those who developed a normal overjet.

**Conclusions:**

The study identified significant relationships between masticatory difficulties and factors associated with age, gender, active caries, and non-nutritive oral habits such as thumb/digit sucking and pacifier use. Younger children and males required more assistance with certain food types. Active caries and thumb/digit sucking habits contributed to general masticatory difficulties and preference for soft foods. The one-year follow-up indicated that improvement in masticatory function varies across age cohorts and were associated with improved occlusal features, such as resolution of anterior crossbite and normalized overjet, reduced pacifier use, and a decrease in the number of decayed teeth.

## Introduction

Mastication is important for properly breaking down food to aid swallowing and allow better absorption of nutrients, and has primarily been investigated in adults. Hatch et al. [1] reported that masticatory performance can be affected by sex, age, the number of functional units, bite force, masseter cross-sectional area and diabetes mellitus. Similarly, Queis [2] found masticatory function to be influenced by occlusal contact area, bite force and rate of chewing.

Satisfactory masticatory function, and thus optimal nutrient absorption, is essential for a child’s growth and development [3]. Investigating masticatory function in children during the years of primary dentition is important as it is a period of significant physiological change and the establishment of life-long habits [4]. Moreover, mastication is considered to be an important stimulus of craniofacial growth and development [5].

Studies have demonstrated that poor masticatory function in children is associated with dental caries [6–8], dental pain-related behaviour [9], younger age [8], bottle feeding [8], high body mass index [7], high frequency of liquid food ingestion [7], less masticatory units [7] and a reduced rate of chewing [2].

However, the association between poor masticatory function and malocclusion remains unclear despite previous research. Gavião et al. [10] noted that pre-school children with open bite and posterior crossbite had poorer food shredding abilities than those with no malocclusion. Similarly, Corrêa et al. [11] also reported reduced mixing ability in children with anterior openbite and Cassi et al. [12] found that both cleft-affected and non-cleft patients with posterior crossbite to have a higher proportion of reverse chewing cycle, compared to control without posterior crossbite., Souto-Souza D et al. [8] also found that masticatory function was negatively impacted by malocclusion. Furthermore, Choi et al. [13] observed that masticatory function worsens with increasing severity of malocclusion. In contrary, Consolação Soares et al. [7] found no relationship between malocclusion and masticatory function in their sample of pre-schoolers. This lack of association was also concurred by Costa et al. [14] and Barrera et al. [15]

Non-nutritive sucking habits such as thumb/digit sucking and pacifier use are common in pre-school children, and have been found to be associated with a higher prevalence of malocclusion [16]. However, whether this can indirectly affect masticatory function through its effect on malocclusion has not yet been comprehensively investigated.

In addition to the conflicting results stated earlier, the effects of occlusal features and non-nutritive sucking habits on masticatory function have not been investigated in the Chinese population which has significantly different dietary habits from Caucasian populations.

Therefore, this study aimed to investigate the direct association of three-dimensional occlusal features on masticatory function, and the indirect association of non-nutritive sucking habits on masticatory function, through its effect on malocclusion, among preschool children in Hong Kong. With the focus sat in evaluating the masticatory function of Chinese pre-school children, three domains were investigated, namely general chewing difficulty, requiring help when eating different food types and increased preference to soft food. General difficulty chewing gave an overview of masticatory function. Requiring help when eating different food types assessed difficulties encountered with foods of different texture and characteristics. For example, the need of help when eating foods containing bones was evaluated, as this type of food contribute a significant portion to the Chinese diet, and the ability to eat them without help was considered as a milestone in Chinese children’s development. Increased preference to soft food was also checked, as it not only reflected poor mastication, but also further hindered the development and subsequent motor training of masticatory muscles, and therefore had a vicious cycle effect on masticatory function [3]. 

In addition to the initial observational investigation, a one-year follow-up was carried out to determine whether improved occlusal features and reduced non-nutritive sucking habits were associated with improved masticatory function.

## Materials and methods

### Samples

Ethical approval was obtained from the Institutional Review Board of the University of Hong Kong/Hospital Authority Hong Kong West Cluster (Reference number: UW14-189).

Using a cluster sampling method, invitations were sent to 17 kindergartens in different geographic locations in Hong Kong run by the same non-governmental organisation (The Hong Kong Society for the Protection of Children). Ten of the 17 kindergartens agreed to participate in the study. These 10 kindergartens were distributed across the three main territories of Hong Kong: Hong Kong Island, Kowloon and the New Territories. Informed consent was obtained from all the participants and their parents or legal guardians involved in the study. Only Chinese participants were included in the study. Children with craniofacial deformities, cleft lips and palate were excluded from the study.

The sample size estimation was based on the primary outcome, which is the association between occlusal features and masticatory function in the cross-sectional investigation. As the probability of masticatory difficulty for each occlusal feature studied had not been previously established, the probability of such events was set at 50% to yield the largest possible sample size. To ensure a 95% CI of 6% (± 3%), a sample of 1,067 was therefore needed.

To account for 20% drop-out due to inability to cooperate during clinical examination, the required sample size was thus 1,333. As the study recruited 1,566 subjects in the cross-sectional survey, the sample size was deemed sufficient.

### Data collection

For the baseline investigation, questionnaires were completed by parents and clinical examinations were conducted by a solo examiner. These data were then used for the cross-sectional analyses. After a one-year observation period without active orthodontic interventions, follow-up data were collected using the same questionnaire and the clinical examination was carried out by the same examiner. These data were subsequently used for the longitudinal investigation.

#### Questionnaire

A universally validated questionnaire has yet to be developed for investigating masticatory function, probably due to the wide diversity in diet extent worldwide. The questionnaire used in the study was customized specifically for investigating masticatory function in Chinese children, and consisted of six questions across three domains as listed in Table 1. Reference was made to Muller’s [17] self-administered *Masticatory Function Questionnaire*, which was used to compare the chewing efficiency of edentulous patients wearing either complete denture or implant overdenture. This self-administered *Masticatory Function Questionnaire* has also been evaluated by Hilassaca-Mamani et al. [18] in 2016, and it was found to have acceptable internal consistency, reliability and validity for investigating the impact of caries on adolescents’ masticatory function. In addition, non-nutritive habits (thumb/digit sucking and pacifier use) and basic demographic data were collected in the questionnaire.

**Table 1:** Questionnaire on masticatory function (the dependent variable)

Domain	Questions
A. General difficulties in chewing	1. Does your child have any difficulty with chewing?
B. Needing help when eating different types of food	2. Do you mince the meat before your child eat? 3. Do you mince the vegetables before your child eat? 4. Do you mince the fruit before your child eat? 5. Does your child need caregiver’s help when eating food containing bones (such as chicken wings and pork ribs) ?
C. Preference for eating soft food	6. Does your child like to eat soft foods?

A 5-point Likert scale was used for the questionnaires, and responses were regrouped into binary outcomes (Difficulties present or not) for statistical analyses.

#### Clinical examinations

Clinical examinations were conducted by a single examiner who recorded all three-dimensional occlusal features and the presence of dental caries. The examiner had more than ten years of clinical experience in orthodontic and pedodontic examinations.

Clinical examinations were carried out in the kindergarten with the child in a supine position under adequate lighting. The child was manipulated into centric occlusion during the examination. A ruler and hand mirror were used to take all measurements. Occlusal features were examined in all three dimensions (sagittal, vertical and transverse), as listed in Table 2.

**Table 2:** Examined variables of children’s three-dimensional occlusal features [19, 20]

Sagittal features	Vertical features	Transverse features
λ **Incisor relationship**, classified as: ***Class I***: lower incisor edges occlude with or lie immediately below the cingulum plateau of upper incisors. ***Class II***: lower incisors edges lie posterior to the cingulum plateau of upper incisors. ***Class III***: lower incisors edges lie anterior to the cingulum plateau of upper incisors. The overjet is reduced or reversed. λ **Anterior crossbite**, classified as: ***Segments***: Both 11, 21 and or more maxillary incisors occluded lingual to mandibular incisors. ***Individual teeth***: *One* maxillary incisors occluded lingual to mandibular incisors. λ **Overjet** : measured from the palatal surface of the mesial corner of the most protruded fully erupted maxillary incisor to the labial surface of the corresponding mandibular incisor. In this study, an overjet of greater than 3 mm was considered increased overjet. λ **Reverse overjet** Measured from the incisal edge of mandibular incisors to the labial surface of the most retruded fully erupted maxillary incisor, which is in anterior crossbite λ **Primary canine relationship**, classified as: ***Class I***: tip of maxillary primary canine tooth is in the same vertical plane as the distal surface of mandibular primary canine ***Class II***: tip of maxillary primary canine tooth is mesial to the distal surface of mandibular primary canine ***Class III***: tip of maxillary primary canine tooth is distal to the distal surface of mandibular primary canine λ **Primary molar relationship**, classified as: ***Flush terminal***: the distal surfaces of the upper and lower second primary molars are in the same vertical plane in centric occlusion ***Distal step***: the distal surfaces of the lower primary second molar is in a posterior relationship to the distal surface of the upper second molar in centric occlusion ***Mesial step***: the distal surfaces of the lower primary second molar is in an anterior relationship to the distal surface of the upper second molar in centric occlusion λ **First molar relationship**, classified as: ***Class I***: mesiobuccal cusp of the upper first molar occludes with the mesiobuccal groove of the lower first molar. Discrepancies of up to half a cusp width either way were also included in this category. ***Class II***: mesiobuccal cusp of the lower first molar occludes distal to the Class I position. ***Class III***: mesiobuccal cusp of the lower first molar occludes mesial to the Class I position. In addition, categories of permanent molars haven’t established occlusion and permanent molars unerupted are included.	λ **Anterior overbite** Coverage of the mandibular incisor by the fully erupted maxillary incisor, classified as: ***< 0, 0–1/2, >1/2***, with reference to the clinical crown height of the mandibular incisors. λ **Posterior open bite** When there are no vertical contacts between upper and lower occlusal surfaces of posterior teeth, both unilaterally or bilaterally.	λ **Crossbite**, recorded on right and left sides, classified as: ***No crossbite present*** ***Crossbite***: When one or more of mandibular canines and molars occluded buccally to the buccal cusps of opposing maxillary teeth molars ***Lingual crossbite***: When one or more of the mandibular primary canines or molars occluded lingually to the lingual cusps of the opposing maxillary teeth

For caries assessment, a grading of 5 or 6 according to the International Caries Detection and Assessment System (ICDAS) II criteria would be assessed as having active caries [21]. Caries assessment was done on a tooth surface level, which was subsequently converted into the number of decayed teeth and caries experience for each participant.

#### One-year follow-up

Follow-up data collected after one year were used for the longitudinal investigation to determine whether age-specific improvements in occlusal features and other factors resulted in improved masticatory function. Improved masticatory function was defined as at least a 1-point improvement on the 5-point questionnaire scale.

### Statistical analysis

The associations between poor masticatory function and different occlusal features, non-nutritive sucking habits and presence of caries at baseline were investigated using chi-squared tests. Multivariate binomial logistic regressions were subsequently carried out, incorporating any significant factors identified by the chi-squared tests.

Longitudinal analysis was performed after the one-year observation period to compare the masticatory function of those who showed improvements in occlusal features, non-nutritive sucking habits and caries with those who did not, again using chi-squared tests.

Statistical Product and Service Solutions® (SPSS®) Statistics 26.0 software was used to perform all of the analyses. Findings were reported as significant when the *p*-value was < 0.05.

## Results

### Sample characteristics

Baseline investigations were carried out for 1,566 preschool children (792 males, 753 females and 21 with unreported sex; cases with unreported sex were excluded from all analyses concerning sex) aged 2–6 (3.67 ± 1.12) years.

At one-year follow-up, a total of 966 Chinese children (499 males, 492 females and 5 with unreported sex; cases with unreported sex were excluded from all analyses concerning sex) aged 2–6 (4.30 ± 0.82) years were included in the study.

The loss of 570 participants at one-year follow-up was due to their graduation from kindergarten.

### Cross-sectional evaluation: association of occlusal features, caries and oral habits with masticatory function

Bivariate tests showed that the following factors were associated with masticatory difficulties: male sex (Questions 1, 2, 5), younger age (Questions 2, 3, 5, 6), presence of active caries (Questions 4, 6), current thumb/digit sucking behaviour (Questions 1, 4, 6), current pacifier use (Questions 2, 3, 4, 5), unilateral permanent molars not in occlusion(Question 3) and an abnormal overjet (< 0 mm or > 3 mm) (Questions 1, 3).

Multivariate analyses were then performed for each of the six questions (Questions 1–6), including any significant factors identified by the respective chi-squared tests, and the results are presented as follows.

#### Domain 1: General difficulties in eating (Question 1)

A significant association was identified between general difficulties with mastication and the presence of active caries. Children with active caries were 2.130-fold more likely to have general difficulties chewing than those without caries [95% CI 1.037–4.373] (binary logistic regression: *p* = 0.040). Moreover, those with ongoing thumb/digit sucking habits were 2.136-fold more likely to experience difficulties eating [95% CI 1.037–4.373] (*p* = 0.020) (Table 3).

**Table 3:** Association between general masticatory difficulty (Question 1) and significant factors identified in chi-squared tests

Variables		Adjusted model		
		*p*-value	OR	95%CI
Gender (Female compared to male)		0.059	0.615	0.371–1.018
Age group	1–2 years old (Compared to 5–6 years old)	0.955	1.024	0.445–2.358
	3–4 years old (Compared to 5–6 years old)	0.903	1.036	0.586–1.831
Active caries present		0.040*	2.130	1.037–4.373
Current thumb/ digit sucking		0.020*	2.136	1.126–4.052

* *p* < 0.05

#### Domain 2: Needing help when eating different types of food (Q2-Q5)

When investigating children’s need for help when eating different types of food, a similar trend was observed across different food types. Sex, age and non-nutritive sucking habits were found to have a significant effect on this need (Tables 4, 5, 6 and 7).

Males were found to be 1.54-fold as likely as females to require meat to be minced prior to eating [95% CI 1.218–1.949] (*p* < 0.001). Moreover, they were 1.295-fold as likely as females to require vegetables to be minced before eating [95% CI 1.022–1.642] (*p* = 0.032), and were 1.347-fold as likely to require help when eating foods with bones [95% CI 1.014–1.788] (*p* = 0.039).

Younger children were more likely to need meat to be minced before eating it. Children aged 1–2 years were 4.860-fold more likely to require their meat to be minced compared to those aged 5–6 years [95% CI 3.184–7.419] (*p* < 0.001). Similarly, children aged 3–4 were 2.158-fold more likely to need their meat minced before eating compared to those aged 5–6 [95% CI 1.646–2.829] (*p* < 0.001). In terms of vegetables, children aged 1–2 were 5.628-fold more likely to need their vegetables minced before eating compared with those aged 5–6 [95% CI 3.676–8.615] (*p* < 0.001), while 3–4 year olds were 2.474-fold as likely to require vegetables to be minced before eating than 5–6-year-olds [95% CI 1.873–3.268] (*p* < 0.001). Younger children were also more likely to need help when eating foods containing bones. Children aged 1–2 years were 2.489-fold more likely to need help than 5–6-year-olds [95% CI 1.386–4.471] (*p* = 0.002), while no significant differences were seen between children aged 3–4 and 5–6 years in terms of requiring help to eat foods containing bones.

Non-nutritive sucking habits were also found to play a significant role in determining masticatory function. Those using pacifier were 2.080-fold more likely to need their vegetables minced prior to eating [95% CI 1.046–4.138] (*p* = 0.037), and were 2.882-fold more likely to need their fruit minced prior to eating [95% CI 1.211–6.857] (*p* = 0.017) compared to those not using a pacifier. Moreover, they were 5.052-fold more likely to need help when eating foods containing bones [95% CI 1.208–21.138] (*p* = 0.019).

The presence of active caries, in contrast, only significantly influenced the need for fruits to be minced before eating. Those with active caries were 2.292-fold more likely than those without caries to require their fruit to be minced before eating [95% CI 1.145–4.590] (*p* = 0.019).

**Table 4:** Association between needing to mince meat before eating (Question 2) and significant factors identified in chi-squared tests

Variables		Adjusted model		
		*p*-value	OR	95%CI
Gender (Female compared to male)		< 0.001***	0.649	0.513–0.821
Age group	1–2 years old (Compared to 5–6 years old)	< 0.001***	4.860	3.184–7.419
	3–4 years old (Compared to 5–6 years old)	< 0.001***	2.158	1.646–2.829
Active caries present		0.500	1.158	0.757–1.771
Current pacifier use		0.261	1.458	0.756–2.815

***p < 0.001

**Table 5:** Association between needing to mince vegetables before eating (Question 3) and significant factors identified in chi-squared tests

Variables		Adjusted model		
		*p*-value	OR	95%CI
Gender (Female compared to male)		0.032*	0.772	0.609–0.978
Age group	1–2 years old (Compared with 5–6 years old)	< 0.001***	5.628	3.676–8.615
	3–4 years old (Compared with 5–6 years old)	< 0.001***	2.474	1.873–3.268
Active caries present		0.986	1.004	0.654–1.540
Current use of pacifier		0.037*	2.080	1.046–4.138
Unilateral Permanent molar in occlusion		0.677	1.001	0.996–1.006
Normal Overjet (0-3 mm)		0.151	0.895	0.769–1.041

* *p* < 0.05; ***p < 0.001

**Table 6:** Association between needing to mince fruit before eating (Question 4) and significant factors identified in chi-squared tests

Variables		Adjusted model		
		*p*-value	OR	95%CI
Gender (Female compared to male)		0.975	1.008	0.632–1.631
Age group	1–2 years old (Compared with 5–6 years old)	0.768	1.121	0.524-2.400
	3–4 years old (Compared with 5–6 years old)	0.647	0.878	0.504–1.532
Active caries present		0.019*	2.292	1.145–4.590
Current thumb/digit sucking		0.283	1.452	0.735–2.871
Current use of pacifier		0.017*	2.882	1.211–6.857
Anterior crossbite		0.977	1.000	0.985–1.015

* *p* < 0.05

**Table 7:** Association between needing help when eating foods with bones (Question 5) and significant factors identified in chi-squared tests

Variables		Adjusted model		
		*p*-value	OR	95%CI
Gender (Female compared to male)		0.039*	0.742	0.559–0.986
Age group	1–2 years old (Compared with 5–6 years old)	0.002**	2.489	1.386–4.471
	3–4 years old (Compared with 5–6 years old)	0.850	0.970	0.704–1.335
Active caries present		0.769	1.081	0.644–1.812
Current use of pacifier		0.027*	5.052	1.208–21.138

* *p* < 0.05; **p < 0.01

#### Domain 3: Preference for eating soft foods (Question`6)

Preference for eating soft foods was associated with the presence of dental caries. Those with active caries were 2.287-fold more likely than those without caries to prefer eating soft foods [95% CI 1.493–3.503] (*p* < 0.001). Moreover, those who sucked their thumb frequently were 1.577-fold more likely than those who did not to prefer soft food [95% CI 1.095–2.270] (*p* = 0.014) (Table 8).

**Table 8:** Association between preference in eating soft foods (Question 6) and significant factors identified in chi-squared tests

Variables		Adjusted model		
		*p*-value	OR	95%CI
Gender (Female compared to male)		0.359	0.898	0.713–1.130
Age group	1–2 years old (Compared with 5–6 years old)	0.268	1.242	0.846–1.823
	3–4 years old (Compared with 5–6 years old)	0.639	1.066	0.815–1.396
Active caries present		< 0.001***	2.287	1.493–3.503
Current thumb/ digit sucking		0.014*	1.577	1.095–2.270

* *p* < 0.05; ***p < 0.001

### Longitudinal evaluation

After a 12-month follow-up period with no active orthodontic interventions, masticatory function was assessed again using chi-squared tests to compare those with improved occlusion features, reduced non-nutritive sucking habits and reduced number of caries to those who did not exhibit these improvements. Basic demographic factors (age and sex) were also included in this repeated survey.

#### Domain 1: General difficulties eating

General masticatory difficulties were reduced in a greater proportion of the 5–6 year old cohort (92.1%) than of the 1–2 year old cohort (43.4%). The 1–2 year old cohort, in turn, experienced a greater reduction in masticatory difficulties than the 3–4 year old cohort (22.9%) (*p* < 0.001). Moreover, general difficulties with chewing were reduced in a larger proportion of those with improved anterior crossbite (100%) than of those without (16.7%) (*p* = 0.047).

#### Domain 2: Needing help when eating different types of food

Concerning the need to mince meat before eating, a larger proportion of those with decreased number of caries (43.8%) experienced a reduced need for mincing than those without (19.8%) (*p* = 0.018).

In terms of eating foods containing bones, those who had reduced pacifier use had a greater reduction in their need for help (39%) than those who did not (24.5%) (*p* = 0.036).

#### Domain 3: Preference for eating soft foods

Those with improved overjet, to within the normal range of 0–3 mm, had a greater reduction in their preference for soft foods (52.2%) than those who had no such improvements (28.6%) (*p* = 0.021).

## Discussion

### Target population

In this study, masticatory function was investigated in Chinese pre-school children. In contrast, most previous studies have focused on Caucasian and Latin-American populations [7, 8]. Most of the Hong Kong population are Chinese and have a significantly different diet from Western populations. For instance, the Chinese more frequently consume foods containing bones. Another major difference is the difference in eating utensils, Chinese always use chopsticks, while Caucasians use knives and forks. This difference mean that foods are less likely to be cut into very small pieces with cutlery before eating, making cutting and chewing essential in mastication.

### Investigation on three-dimensional features of occlusion

While previous studies [7, 8] have investigated the effect of different locations of malocclusion (that is, whether in the anterior or posterior part of the dentition) on masticatory function, the current study comprehensively investigate the effects of individual occlusal features on mastication, allowing a systematic identification of significant contributing factors. This is important as occlusal features may specifically affect different domains of masticatory function. For instance, in this study, an improvement in anterior crossbite led to improved general masticatory function, while improved overjet led to a reduced preference for soft foods. Toro et al. [22] found that Peer Assessment Rating (PAR) index did not serve as a reliable predictor of masticatory performance, despite finding that malocclusion negatively affected chewing. This could be due to the inclusion of overall but unspecific occlusal features in the PAR index, given that specific occlusal features alone, such as anterior crossbite and abnormal overjet, influenced masticatory function in the present study.

### Factors associated with poor masticatory function

We found that certain occlusal features, non-nutritive sucking habits, male sex, younger age and the presence of dental caries were significantly associated with poor masticatory function.

#### Sex

We found that female participants required less help when eating meat, vegetables and foods containing bones. This might be because girls develop superior self-help skills at a younger age than boys do, as reported by Moser [23]. In contrast, Barrera et al. [15] found limited sex-specific differences in masticatory performance. These contrasting results are probably because self-help skills are particularly relevant for the Chinese diet, such as developing the skill to identify and remove bones when eating foods like chicken wings and pork ribs.

#### Age

We noted a reduction in the amount of help needed when eating meat, vegetables and foods containing bones with increasing age.

In the longitudinal investigation, 5–6-year-olds showed the greatest improvements in chewing, compared with 1–2- and 3–4-year-olds.

These results agree with previous studies that have suggested masticatory function improves with age [8, 31]. Toro [22] suggested that age-based differences in masticatory performance relate to increasing body size with age. Other age-related factors have also been proposed, such as the shedding of deciduous teeth in late mixed dentition negatively affecting masticatory function [15].

An interesting finding of this study was the lack of any significant differences between children aged 3–4 years old and those aged 5–6 years old in terms of their need for help when eating foods containing bones. Based on these findings, 4 years old appears to be a developmental milestone when Chinese children develop the ability to eat foods with bones with minimal assistance.

#### Occlusal features

##### Sagittal features

An abnormal overjet was found to be associated with an increased need to mince vegetables before eating them. However, when logistic regressions were performed, the significant effect of an abnormal overjet on masticatory function was lost, suggesting that other factors, such as age and caries, may contribute more significantly to masticatory function.

In the longitudinal investigation, those with improved anterior crossbite experienced improved general chewing ability. Those with improved overjet, to within the normal range of 0–3 mm, showed a reduced preference for soft foods.

##### Vertical features

Occlusal features, such as permanent molars not contacting, were found to be associated with an increased need to mince vegetables before eating them in a bivariate analysis of the cross-sectional survey data. This finding agrees with a previous study by Owens et al. [24], wherein Class I cases had larger contact areas, followed by Class II and then Class III cases, with masticatory performance ranking in the same order. In a systematic review by Magalhães et al. [25] in 2010, malocclusion was found to lead to decreased masticatory performance, especially when associated with reduced occlusal contact.

However, when binary logistic regressions were performed, the significant effect of molars not contacting was lost. This may be because the increase in contact area brought about by the eruption of an additional pair of permanent molars was relatively small, leading to an insignificant result in the multivariate analysis.

Therefore, results from the current study confirmed that improvements in specific occlusal features can lead to improved masticatory function. This aligned with recent studies on the topic, which focused on whether improved occlusion bought by orthodontics can lead to better masticatory function. Gameiro et al. [26] found that masticatory performance was significantly improved in the malocclusion group by fixed appliance therapy. The meta-analysis by Zhan et al. [27] in 2023 reported improved electrical activity of masticatory muscles after orthodontic treatment. Concerning the correction of specific malocclusion, Goncalves et al. [28] found correction of anterior openbite and posterior crossbite in children promoted functional alteration in electromyographic activity of masseter and temporalis muscles. With regard to different orthodontic modalities, Ashok et al. [29] and Piancino et al. [30] found functional appliance effectively improve masticatory efficiency in cases with retrognathic mandible and cases with deep bite respectively. Orthognathic surgery was also found by Celakil et al. [31] to improve masticatory performance. In contrary, Henrikson et al. [32] reported that adolescent girls with Class II malocclusion corrected orthodontically showed similar chewing efficiency to girls with untreated Class II malocclusion. In view of this and other conflicting findings, the systematic review by Alshammari et al. [33] concluded it was difficult to support or deny the influence of both malocclusion and subsequent orthodontic treatment on the masticatory performance in children, and further research should be conducted to shed light on this controversial issue.

Finally, Grabowski et al. [34] suggested that orthodontic and functional symptoms in early dentition can result in malocclusion later in development. This bidirectional relationship should be kept in mind when investigating masticatory function in young children, both to the researchers and the clinicians.

#### Non-nutritive sucking habits

Thumb/digit sucking adversely affected masticatory function, with increased general chewing difficulty and increased preference for soft foods. Pacifier use was similarly associated with an increased need for help when eating vegetables, fruits and foods containing bones. In the longitudinal evaluation, those with reduced frequency of pacifier use required less help when eating foods containing bones. These findings were expected, as non-nutritive sucking habits have been found by Ling et al. [35] to contribute to malocclusions such as anterior open bite and reduced overbite. This can negatively affect masticatory function. Additionally, reducing non-nutritive sucking behaviours may promote the development of self-care skills, again improving masticatory function.

#### Dental caries

Dental caries adversely affected masticatory function in terms of general difficulties eating, difficulty in eating fruits and increased preference for soft foods. In the longitudinal follow-up, reduction in number of active caries was associated with a reduced need to mince meat before eating. Sensitivity and pain during eating are likely to occur with cavities, defined as grade 5 or 6 according to the ICDAS II criteria, and may prevent a child from eating efficiently or comfortably. These findings agree with previous studies that have reported that children with early childhood caries have significantly worsened masticatory performance [6–8, 36]. Linas et al. [37] found that children with early childhood caries showed reduced chewing frequency and more frequently refused carrots. However, the author also noted that these caries-affected children developed alternative behavioural strategies to overcome feeding difficulties.

### Validity of self-administered questionnaires for investigating masticatory function

Besides the use of the *Masticatory Function Questionnaire* by Muller and Hilassaca as stated earlier, self-administered questionnaires had also been used to examine the number of functional teeth required for satisfactory masticatory efficiency in a similarly large-scale Japanese study by Ueno et al., [38] involving 2,164 subjects.

Self-administered questionnaire data and masticatory function had also been reported as being positively correlated. Marquezin [39] found that poor masticatory performance was significantly correlated with an increased Nordic Orofacial Test-Screening (NOT-S) score. In a study by Linas et al. [37] in 2018, two self-administered questionnaires, the Early Childhood Oral Health Impaction Scale (ECOHIS) and the NOT-S, yielded higher scores for children with early childhood caries who had poor masticatory function, as evidenced by reduced chewing frequency and refusal of certain foods. Choi et al. [13] also showed high correlation between the self-reported questionnaire *Food Intake Ability* and the mixing ability index, which was a colorimetric method to subjectively investigate masticatory performance. A similarity between the *Food Intake Ability* questionnaire and the questionnaire used in our study was that food type specific to the culture was purposely included, for example, dried cuttle fish and white radish kimchi was included in the *Food Intake Ability* questionnaire for their Korean sample.

We therefore believed that the use of questionnaire customized according to the diet culture to evaluate masticatory function in a large cohort of pre-school children was reasonable. However, it would be beneficial to further validate the questionnaire in the future by performing specific masticatory function tests.

### Limitations

This study had some limitations. First, cluster sampling of kindergartens across different regions in Hong Kong was carried out to eliminate the geographical bias, however, this form of sampling is not as robust as completely randomised sampling, as biases in socio-economic status might still be present.

Second, only self-reported questionnaires were used to assess chewing difficulties, without conducting masticatory performance test. A previous study investigating the association of malocclusion and caries with masticatory function used the masticatory performance test, in which the median particle sizes of silicone samples were recorded after 20 chewing cycles [7]. This method was not used in the current study due to the difficulty of conducting the masticatory performance test with the large sample size in an outreach environment. Also, the sensitivity of such a masticatory performance test had been questioned due to the artificial nature of the chewable test material [36].

## Conclusions

The study identified significant relationships between masticatory difficulties and factors associated with age, gender, active caries, and non-nutritive oral habits such as thumb/digit sucking and pacifier use. Younger children and males required more assistance with certain food types. Active caries and thumb/digit sucking habits contributed to general masticatory difficulties and preference for soft foods. The one-year follow-up indicated that improvement in masticatory function varies across age cohorts and were associated with improved occlusal features, such as resolution of anterior crossbite and normalized overjet, reduced pacifier use, and a decrease in the number of decayed teeth.

## Data Availability

The data that support the findings of this study are available from the corresponding author, Y.Y., upon reasonable request.

## Abbreviations

ICDAS: International Caries Detection and Assessment
SPSS®: Statistical Product and Service Solutions®
PAR: Peer Assessment Rating
NOT-S: Nordic Orofacial Test-Screening
ECOHIS: Early Childhood Oral Health Impaction Scale
